# ITN—VIROINF: Understanding (Harmful) Virus-Host Interactions by Linking Virology and Bioinformatics

**DOI:** 10.3390/v13050766

**Published:** 2021-04-27

**Authors:** Winfried Goettsch, Niko Beerenwinkel, Li Deng, Lars Dölken, Bas E. Dutilh, Florian Erhard, Lars Kaderali, Max von Kleist, Roland Marquet, Jelle Matthijnssens, Shawna McCallin, Dino McMahon, Thomas Rattei, Ronald P. Van Rij, David L. Robertson, Martin Schwemmle, Noam Stern-Ginossar, Manja Marz

**Affiliations:** 1 RNA Bioinformatics and High-Throughput Analysis, Friedrich Schiller University, 07743 Jena, Germany; winfried.goettsch@uni-jena.de; 2 Department of Biosystems Science and Engineering, ETH Zurich, Mattenstrasse 26, 4058 Basel, Switzerland; niko.beerenwinkel@bsse.ethz.ch; 3 Institute of Virology, Helmholtz Centre Munich and Technical University Munich, Ingolstädter Landstraße 1, 85764 Neuherberg, Germany; li.deng@helmholtz-muenchen.de; 4 Institut für Virologie und Immunbiologie, Julius-Maximilians-Universität Würzburg, 97078 Würzburg, Germany; lars.doelken@uni-wuerzburg.de (L.D.); florian.erhard@uni-wuerzburg.de (F.E.); 5 Theoretical Biology and Bioinformatics, Science for Life, Utrecht University, Hugo R. Kruytgebouw, Padualaan 8, 3584 CH Utrecht, The Netherlands; bedutilh@gmail.com; 6 Institute of Bioinformatics, University Medicine Greifswald, 17475 Greifswald, Germany; lars.kaderali@uni-greifswald.de; 7 MF1 Bioinformatics, Robert Koch-Institute, 13353 Berlin, Germany; kleistm@rki.de; 8 CNRS, Architecture et Réactivité de l’ARN, Université de Strasbourg, UPR 9002 Strasbourg, France; r.marquet@ibmc-cnrs.unistra.fr; 9 Department of Microbiology and Immunology, Katholieke Universiteit Leuven, Herestraat 49 Box 1040, 3000 Leuven, Belgium; jelle.matthijnssens@kuleuven.be; 10 Department of Neuro-Urology, Balgrist University Hospital, University of Zürich, Forchstrasse 340, 8008 Zürich, Switzerland; shawna.mccallin@gmail.com; 11 Institute of Biology, Freie Universität Berlin, Schwendenerstr. 1, 14195 Berlin, Germany; dino.mcmahon@fu-berlin.de; 12 Division of Computational Systems Biology, Centre for Microbiology and Environmental Systems Science, University of Vienna, Althanstraße 14, 1090 Vienna, Austria; thomas.rattei@univie.ac.at; 13 Department of Medical Microbiology, Radboud Institute for Molecular Life Sciences, Radboud University Medical Center, P.O. Box 9101, 6500 HB Nijmegen, The Netherlands; ronald.vanrij@radboudumc.nl; 14 MRC, University of Glasgow Centre for Virus Research (CVR), 464 Bearsden Road, Glasgow G61 1QH, UK; David.L.Robertson@glasgow.ac.uk; 15 Institute of Virology, Medical Center—University of Freiburg, Hermann-Herder-Strasse 11, 79104 Freiburg, Germany; martin.schwemmle@uniklinik-freiburg.de; 16 Department of Molecular Genetics, Weizmann Institute of Science, Rehovot 7610001, Israel; noam.stern-ginossar@weizmann.ac.il; 17 FLI Leibniz Institute for Age Research, 07745 Jena, Germany

**Keywords:** bioinformatic, virus, virology, virus host interaction

## Abstract

Many recent studies highlight the fundamental importance of viruses. Besides their important role as human and animal pathogens, their beneficial, commensal or harmful functions are poorly understood. By developing and applying tailored bioinformatical tools in important virological models, the Marie Skłodowska-Curie Initiative International Training Network VIROINF will provide a better understanding of viruses and the interaction with their hosts. This will open the door to validate methods of improving viral growth, morphogenesis and development, as well as to control strategies against unwanted microorganisms. The key feature of VIROINF is its interdisciplinary nature, which brings together virologists and bioinformaticians to achieve common goals.

## 1. Introduction

**Figure 1:**
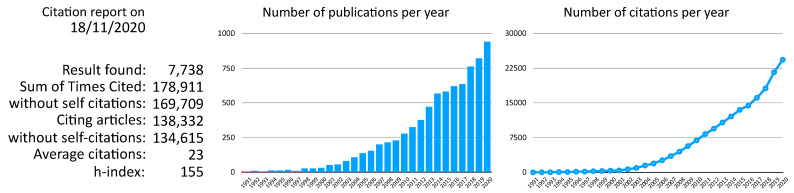
Citation report extracted from Thomson Reuters Web of Science for “((virus) OR (viral)) AND ((bioinformatics) OR (computational))”.

The Innovative Training Network (ITN) is one of the Marie Skłodowska-Curie Actions of the European Commission, which aims to train a new generation of creative, entrepreneurial and innovative early-stage researchers, able to face current and future challenges and to convert knowledge and ideas into products and services for economic and social benefit. Within the ITN VIROINF, we aim to understand virus-host interactions, by bridging virology and bioinformatics being an emerging field, see Figure 1. We will train a new generation of virologists and bioinformaticians to develop novel strategies to better understand, treat, and control viral diseases in interaction with hosts, in addition to understanding the broader ecology and evolution of viruses in the context of their hosts. VIROINF thus aims to move beyond bioinformatical developments for virus research (which are often simply adopted from non-viral research) to focus on the major open questions in virology. We will tackle this by exploiting the great potential of systems biology methodology and integrative bioinformatics analysis in a multidisciplinary ITN. All projects consist of pairs of researchers comprised of one virologist and one bioinformatician, who will collaborate to study virus-host interactions and virus evolution.

## 2. The Research Programme

**Figure 2:**
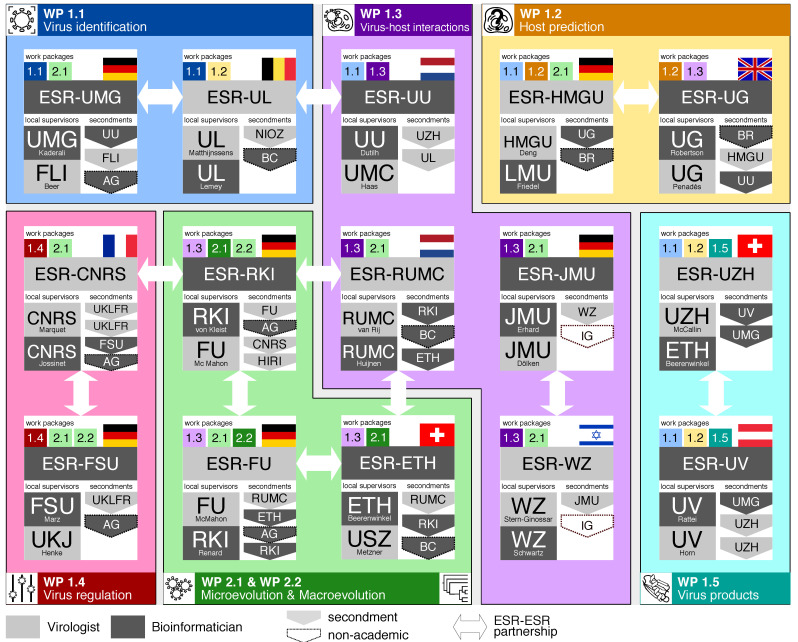
The VIROINF network of early-stage researchers (ESRs), local supervisors, ESR-ESR partnerships, and secondments. Each ESR is either bioinformatician or virologist and forms a partnership with another ESR of the other scientific field (white arrows). Each project interacts with at least 5 other projects. Locally two supervisors of different background train the ESR to guarantee a truly interdisciplinary work. An ESR focuses on one working package and is associated to at least one more working package (coloured numbers per ESR). Each ESR has at least two secondments. AG—AllGenetics; BC—Baseclear; BR—BioRelate; FLI—Friedrich-Löffler-Institut; HIRI—Helmholtz Institute for RNA-based Infection Research; IG—InfectoGnostics Research Campus; NIOZ—NL Institute for Sea Research; UKLFR—Universitätsklinikum Freiburg; UU—University Medical Center Utrecht.

The main themes of VIROINF are: (i) Modelling virus-host interactions, including virus identification, host prediction, virus-host interactions, virus regulation, and virus products; and (ii) Modelling virus evolution in hosts, including microevolution (virus quasispecies) and macroevolution (natural selection of viruses, see Figure 2).

**Figure 3:**
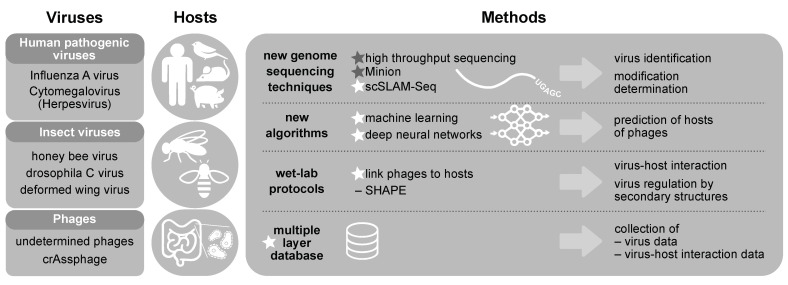
In VIROINF we aim to understand virus-host-interactions for viruses infecting human, bird, mouse and pig (Influenza A virus, Cytomegalovirus), insects (honey bee virus, nora and drosophila C virus, deformed wing virus) and microbiota (mainly phages). This is complemented by the usage of new genome sequencing techniques, new algorithms, wet-lab protocols and the establishment of a multiple layer database. Stars indicate methods developed. Friedrich Schiller University (FSU) runs the sequencing core facility and developed several virus-specific protocols for sequencing on Illumina and Minion platforms.

After the foundation of the European Virus Bioinformatics Center (EVBC) in Jena in 2017, a broad range of applied bioinformaticians and virologists met to guide developments in virus bioinformatics. The interactions between the members resulted in the identification of a broad range of opportunities for collaboration between the research groups. In order to create a framework for collaboration between these research groups we propose the implementation of the VIROINF ITN [1,2]. The research training programme tackles major bioinformatical and virological challenges: the power of new genome sequencing technologies, associated with new tools to handle “big data”, provide unprecedented opportunities to address fundamental questions in virology. We emphasise that many of the common questions raised in virology require specific bioinformatics support [3] and require the combined expertise of both bioinformaticians and virologists. Important fundamental questions in virus-host biology remain unanswered and the underpinning bioinformatical tools required to address many of these questions are currently underdeveloped. The strategy of the network is therefore to develop parallel lines of bioinformatical research, to address a key set of concrete questions in virus research (see Figure 3).

## 3. Modelling Virus-Host Interactions

This part of the ITN aims at developing fundamental bioinformatical tools for modelling virus-host interactions and understanding these interactions mechanistically. In order to describe virus-host interactions, interacting viruses need to be identified and hosts need to be determined.

### 3.1. Virus Identification

Despite large ongoing efforts to identify viruses in humans, animals and the environment, there are still multiple challenges remaining in the identification of novel viruses. These challenges concern both the wet-lab as well as the bioinformatical side. On the wet-lab side, divergent viruses can now be identified from an infected sample *en masse*, but different research groups use different protocols to analyse these samples and prepare them for sequencing. All the current approaches result in relative distributions, which lack absolute quantitation. Sample preparation, methods and standardisation are prerequisites for meaningful and comparative work. On the bioinformatical side, the most frequently used similarity-based virus identification approaches are largely limited by available databases, which are incomplete and often poorly annotated. VIROINF aims to set up a multiple layer database for phages and their hosts, which with the help of the European Virus Bioinformatics Center will be performed in the first months of VIROINF.

VIROINF aims to quantify viruses by the following four approaches: (i) spiking in known amounts of selected viruses into a sample as internal controls; (ii) counting virus particles using flow cytometric approaches in collaboration with Corina Brussaard from the Netherlands Institute for Sea Research; (iii) test Minion sequencing technology on unamplified extracted viral nucleic acids, and (iv) NGS-based quantification developed in collaboration with non-academic partner Baseclear. The novel wet-lab approaches will be combined with newly developed advanced bioinformatical tools to identify viruses from metagenomics datasets (ESR-UMG), using sequence dependent and independent approaches. Motif searches, k-mer frequencies, and genome organisation are de novo approaches that will complement homology-based methods, together allowing the detection of known and unknown viruses, yielding a comprehensive detection pipeline of all types of viruses ranging from well-studied human pathogens to completely novel bacteriophages. A range of available tools for taxonomic classification will be combined into a rapid pipeline that will be used from many beneficiaries: ESR-FU, ESR-HMGU, ESR-JMU, ESR-ETH, ESR-RKI, ESR-UZH and ESR-UL. These tools will be made available in the public domain by VIROINF. Together, these developed wet-lab and bioinformatical approaches will be used by ESR-UL to provide an in depth in silico and in vivo characterization of the bacteriophage population infecting the most important members of the honeybee gut microbiota, as well as studying their role in honey bee health and development.

**ESR-UL—Development of more quantitative viral metagenomics approaches to investigate the role of bacteriophages in the honey bee gut and their effects on health and development.** Very few is known about the role of the gut virus population (the virome) in health and disease of honey bees. Recently the NetoVIR protocol was developed to purify viral particles from biological samples, before deep sequencing using Illumina NGS technology [4]. Using this approach, a diverse set of bacteriophages associated with honey bees was described. However, this type of research is hampered by the inherent introduction of biases in the viral composition after viral purification and random amplification before Illumina sequencing. Furthermore, the interpretation of the roles of identified novel bacteriophages in health and diseases, is hampered by many factors including the lack of good sequence databases and knowledge about their bacterial host. In the current project, we will: (i) test and validate different approaches to obtain a more quantitative virome protocol; (ii) Link novel bacteriophages to their host using bacterial 16S data, as well as novel tools developed in the framework of VIROINF and (iii) use these optimized wetlab and in silico protocols to study the role of bacteriophages in honey bee health, using in vitro and in vivo approaches. For the first aim we will use: (a) approaches to spike in known amounts of viruses into the samples as internal controls, (b) methods to accurately count virus particles by flow cytometry (in collaboration with NIOZ) and (c) test Minion sequencing technology on unamplified extracted viral nucleic acids. For the second aim we will collaborate closely with ESR-UMG, ESR-UU and Baseclear for the improvement of the existing bioinformatics analyses and interpretation of the viral NGS data. Their expertise in bacteriophage and eukaryotic virus identification from metagenomics datasets, respectively, and the tools they will develop will be very important for the success of this project.

**Figure 4:**
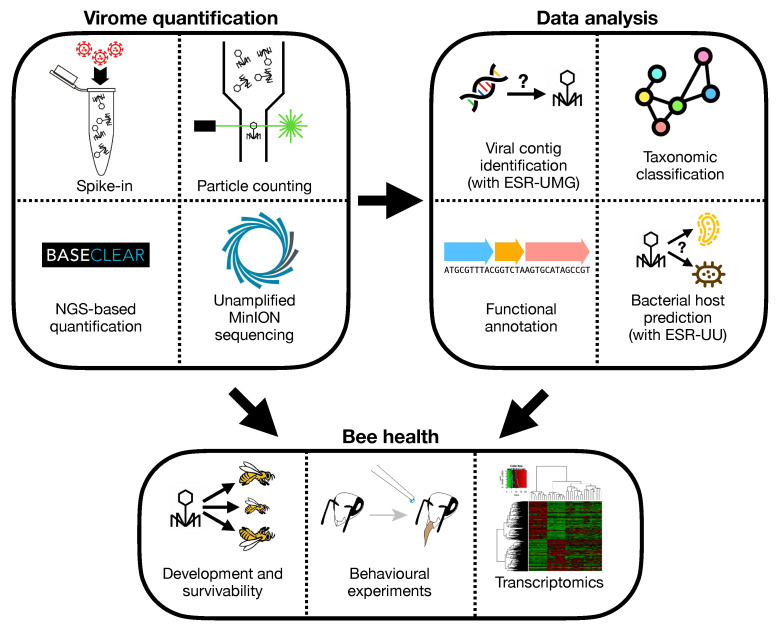
Workflow to decipher bee health by quantification of viral metagenomics of gut.

The third objective of this project is to provide an in depth in silico and in vivo characterization of the bacteriophage population infecting the most important members of the honeybee gut microbiota, as well as studying their role in honey bee health and development. Both the bacteriome (16 s) and the virome (shotgun) inhabiting the digestive tract of these honey bees will be characterized. Furthermore, bacteria will be isolated and characterized from the crop, midgut and hindgut of the collected honeybees, and finally these bacterial isolates will be used to isolate bacteriophages followed by their genome sequencing and functional gene annotation. Depending on the available time, we may also start with inoculation experiments of life honey bees (see Figure 4).

**ESR-UMG—A machine learning pipeline to identify and characterise novel viruses in metagenome data.** The wide use of deep sequencing technology in biology and medicine has led to huge publicly available sequencing data bases, offering novel insights into the genetic and genomic makeup of life. Similarly, metagenome sequencing projects offer a deep view into microbiomes. Only a minuscule fraction of the virome has so far been identified; however, many hitherto unknown viral sequences lie hidden in already available data. The objective is to develop efficient computational pipelines to search for novel viruses in genome or metagenome sequencing data, as well as in public DNA and RNA sequencing databases such as NCBI Genbank/SRA. Template-based search strategies will be developed to search for unknown viruses that are related to a known virus family which is used as search template [5]. We will develop neuronal-network based approaches to learn characteristic features of viral sequences, and compare their performance to support vector machines with a suitable string kernel as well as other supervised machine learning approaches. Two different problems will be addressed: (i) Identifying viral sequences or integrated viral subsequences after genome assembly, versus (ii) their identification directly at the level of raw sequencing reads. Efficient hashing techniques, k-mer string matching and rapid filtering based on suffix arrays will be used to speed up the search through large databases. In addition to the identification of viral sequences, we will also train the algorithms to identify the most likely host(s) based on assembled viral sequences, and to predict pathogenicity. The developed tools will be tested on NCBI Genbank data and using metagenome data from the Study of Health in Pomerania (SHIP), a population-based study carried out at University Medicine Greifswald (see Figure 4).

### 3.2. Host Prediction

Hosts of viruses infecting higher eukaryotes have been well described in the past, whereas the specific hosts of bacteria-infecting viruses are usually unknown. The aims of VIROINF are to investigate how viruses of Bacteria or Archaea (bacteriophages) are functionally linked to each other and to their unicellular hosts. This is crucial for understanding how phages impact the microbiome as they act as key regulators of bacterial population size [6], and can be introduced into natural systems in order to influence species presence. Evidence to date indicates this relationship tends to be highly specific with phages targeting a limited number of bacterial hosts.

VIROINF will, for the first time, address this topic using a combined experimental and computational approach: ESR-HMGU develops methods to experimentally link phages to their hosts as described by Li Deng [7], as well as screen and isolate phages that infect specific hosts at the single phage-particle level. A multiple layer database will be generated by the two partners ESR-HMGU and ESR-UG using the above developed methods containing (i) phage genomes that infect selected hosts; (ii) viral-tagged metagenomes of phages infecting the same hosts; (iii) community co-occurrence of microbiomes and viromes from the same ecosystems. This data will be complemented by text-mined literature data from company partner BioRelate, and data from ESR-UU. Combined this will comprise many 10,000 s of interactions. This dataset will be available for the benchmarking of bioinformatics approaches for inferring host specificity (ESR-UG). Next, ESR-UG will develop machine learning tools to link newly identified bacteriophages to their bacterial host using a range of signals including CRISPR sequences and prophages in bacterial genomes (obtained by ESR-HMGU), k-mer frequencies/genome composition signals, protein domains etc. based on approaches, such as random forests and neural networks/deep learning. Both methods are emerging as two of the most successful machine learning approaches, due to their ability to discover non-linear signals in data and their relative insensitivity to biases in training datasets. This is needed because known viruses represent a small minority of all viruses, and VIROINF aims to create a scalable tool that predicts a host at the optimal level for a given virus. The knowledge of different levels of virus-host co-evolution that will be generated in WP 2.1 will be applied here. The aim will be to infer networks of probable virus-host relationships at species level. To study virus-host specificity at the strain level we will focus on the bacteriophage that infect *Staphylococcus aureus*. Phages are important mediators of bacterial virulence and hence impact human health. Understanding how bacterial gene content is influenced by this lateral gene transfer mechanism is important for understanding how viruses impact both the specific bacteria and the human host [8]. The tools for virus-host predictions generated in this WP 1.2 are used by ESR-UZH, ESR-UU, ESR-FU, ESR-UL, ESR-UMG, and ESR-RKI.

**Figure 5:**
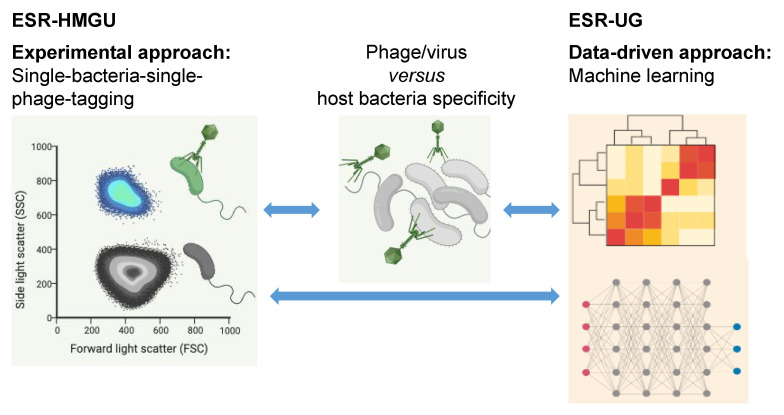
Link between experimental and data-driven approach of ESR-HMGU and ESR-UG to investigate phage-host relationship.

**ESR-HMGU—Bacteriophage–host relationships prediction.** This project seeks to understand how (bacterio)phages are functionally linked to their Bacteria or Archaea hosts both using experimental and bioinformatics approach. Phages identified using cultivation independent viral metagenomic sequencing approach lack sequence similarity to any of previously sequenced ones and can hardly be assigned to their hosts. Different methods exist to predict hosts for phage sequence, e.g., single or multiple features (co-abundance, sequence homology, similarity to other phages or sequence composition similarity between phages and their hosts), or using these features in machine learning models. Therefore, we aim to integrate features of our unique experimental identified, host-linked viral metagenomic data in a deep learning model to improve the prediction performance. Particular attention will be given to phages of anaerobic bacteria which are underrepresented comparing to those infecting aerobic bacteria [9]. ESR-HMGU will develop a modified version for experimentally linking phages to their anaerobic hosts based on the previously published culture independent viral-tagging (VT) method for aerobic bacteria-phage pairs [7,10,11]. In this single cell viral tagging approach, virions from an environmental sample are fluorescently labeled through DNA staining and upon them infecting host cells, the infected host is labelled (see Figure 5). This is followed by single cell sorting via FACS of the infected hosts together with the infecting phages.

**ESR-UG—Computational prediction of virus-host interactions in the microbiome.** Virus-host interactions are highly specific as viruses (including bacteriophages/phages) have co-evolved with the systems they infect and depend on for replication. As a consequence, phylogenetic relatedness and shared genomic properties of host species are a strong predictors of host infectivity. However, our knowledge of host relationships remains sparse and is unknown for many viruses and phages, particularly for metagenomically assembled data. In this project, existing resources and genomic signals will be leveraged to infer missing/unobserved and probable hosts, e.g., where infection has not been observed due to host immunity. Machine learning models will be trained on this data to predict virus-host/ phage-bacteria interactions at the species and strain levels (see Figure 5). Specific objectives are: (a) Construction of bipartite networks of observed virus-host species links based on databases such as Virus-Host DB and MVP, text-mined literature data from company partner BioRelate, and data from ESR-UU. Combined this will comprise many 10,000 s of interactions from observed phage-bacteria interactions, and inferred from prophage and CRISPR- Cas associated viral sequences embedded in host genomes. (b) Quantification of the co-phylogenetic signal using the software Jane to test the preferential host-switching model in bacteria and to determine the extent of co-speciation versus host-switching. This will give information on how stable phage-host interactions are at the species level. (c) Application of combined support-vector machine (SVM) models to virus-host prediction by using different representations of the virus genome information: nucleotide sequence, amino acid sequence, amino acid properties and protein domains. (d) Analysis of virus-host links at the strain level. To achieve this objective we will use the bacteria Staphylococcus aureus (in collaboration with José Penades at Imperial College London) as a model system to study strain level phage-host interaction prediction in the context of CRISPR-Cas immunity and host pan-genomes.

**ESR-UU—Functional inferences from colinear crAssphage genomes.** Viruses related to crAssphage, a bacteriophage discovered by us in 2014 [12], are abundant in the human intestinal tract, human-associated environments, and have been found in diverse non-human primates [13].

**Figure 6:**
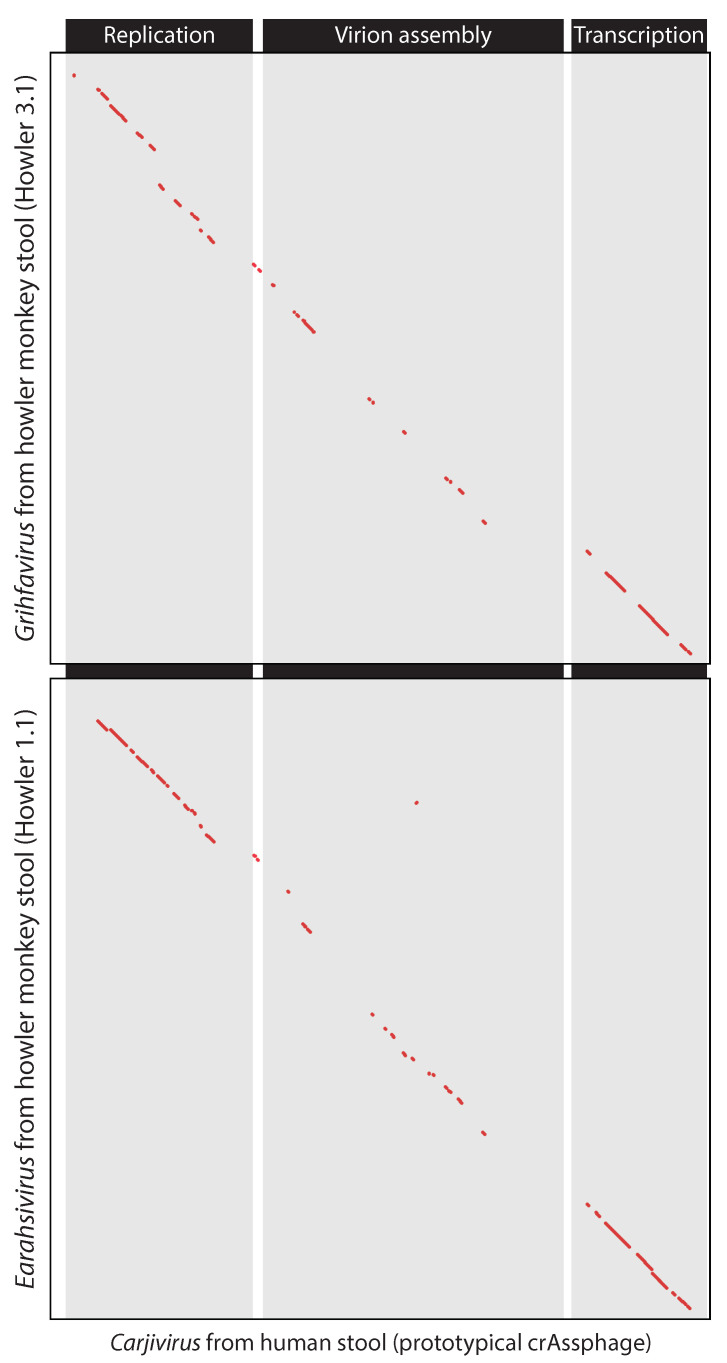
Dotplots showing genomic colinearity between the prototypical crAssphage, originally identified in human stool (subfamily Crudevirinae, genus Carjivirus) and two crAss-like phages found in howler monkeys (top: subfamily Obtuvirinae, genus Grihfavirus, bottom: subfamily Crudevirinae, genus Earahsivirus) that were recently described by Edwards et al. [13]. Taxonomic classifications according to the ICTV Crassvirales Study Group [15]. Genomic regions encoding genes involved in replication, virion assembly, and transcription are indicated according to the annotations by Yutin et al. [16].

We computationally predicted that crAss-like bacteriophages infect *Bacteroidetes* hosts and this was confirmed when the virus was cultured [14]. Strikingly, amplification of the bacteriophages did not impair the host, but the molecular mechanisms involved remain unknown because the proteins involved in host interaction remain unknown (see Figure 6). ESR-UU will investigate the function and evolution of proteins in crAss-like viruses. We recently discovered that crAss-like phages have been genomically co-linear for millions of years [13]. We will exploit this and other signals from fundamental processes in genome evolution to predict host-interaction proteins and further our understanding of virus-host interaction, in particular of crAss-like bacteriophages.

### 3.3. Virus-Host Interactions

VIROINF aims to develop new tools to study virus-host interactions across important virus and host species (see Figure 3).

During productive infection, viral proteins and RNAs interact with cellular pathways to reprogram their hosts cells for efficient virus replication and immune evasion. VIROINF will study virus-host interactions in a specific context to be used afterwards generically: ESR-JMU will study the modulation of hosts cells upon cytomegalovirus infection using metabolic RNA labeling coupled to single cell RNA sequencing (scSLAM-seq) [17]. A new approach called Heterogeneity sequencing enables measurement of every cell at two time points and to infer functional genetic interactions by modeling transcriptional activity of individual cells in response to a perturbation based on their pre-perturbation expression heterogeneity. This technique will be used to study factors that determine the infection outcome in CMV infection, to analyze their conservation in the human system and the mouse model and investigate conserved reprogramming mechanisms. New computational tools will be developed for the integrative analysis of the big heterogeneous data sets alongside with a computational model of CMV induced reprogramming of the regulatory network.

**Figure 7:**
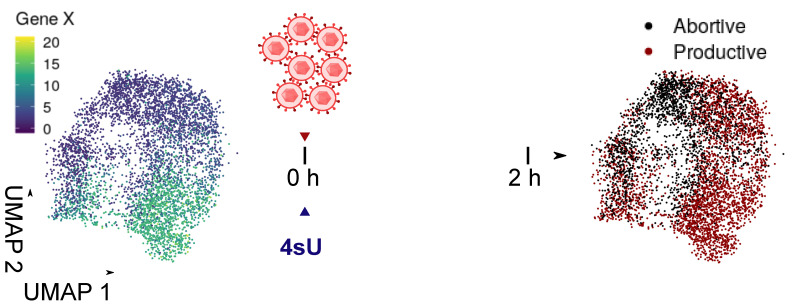
Principle of functional genomics using scSLAM-seq. A heterogeneous cell population is infected and newly transcribed RNA is concomitantly labeled with 4 sU. After 2 h, the RNA from single cells is sequenced and pre-existing RNA (reflecting the cell state prior to infection) and newly synthesized RNA (infection outcome) is separated using the SLAM-seq approach. Functional interactions are inferred by modeling the outcome using the cell states prior to infection.

**ESR-JMU—Conservation of regulatory elements and effector mechanisms in lytic and latent cytomegalovirus infection.** The human cytomegalovirus (HCMV) is a herpesvirus prevalent worldwide responsible for life-threatening infections in individuals with an impaired or immature immune system. During the first 1–2 h after virus entry into a cell, a decision is made whether this results in lytic, latent or abortive infection. Previous single cell RNA-seq (scRNA-seq) approaches did neither provide the necessary temporal resolution nor sensitivity to analyse this crucial phase of virus-host interaction. To overcome these limitations, we recently developed single-cell SLAM-seq (scSLAM-seq, see Figure 7), which combines metabolic RNA labelling with thiol-(SH)-linked nucleotide conversion sequencing (SLAM-seq) and single cell sequencing (scRNA-seq). scSLAM-seq visualised dynamic changes in transcriptional activity during the first 2 h of lytic murine CMV (MCMV) infection. By inferring expression profiles prior to infection for each cell, scSLAM-seq provides unique opportunities to identify factors that influence on the infection outcome. As a proof of concept, we predicted the infection efficiency in murine fibroblasts based on cell cycle and dose of infection with unprecedented accuracy [17]. By emplyoing this technique for human CMV infection we will (a) identify (viral and host) key factors that determine the infection outcome, (b) infer the context dependent regulatory network of the host cell and its changes induced by CMV infection, (c) analyse the evolutionary conservation of these mechanisms between MCMV and HCMV and (d) assess silencing of residual lytic and establishment of true latent viral gene expression.

This will clarify how HCMV reprograms the latently infected cell while avoiding detrimental immune recognition by cytotoxic T cells. To pursue these goals, the we will continue developing our established software tools [18] for (sc)SLAM-seq data analysis.

**Figure 8:**
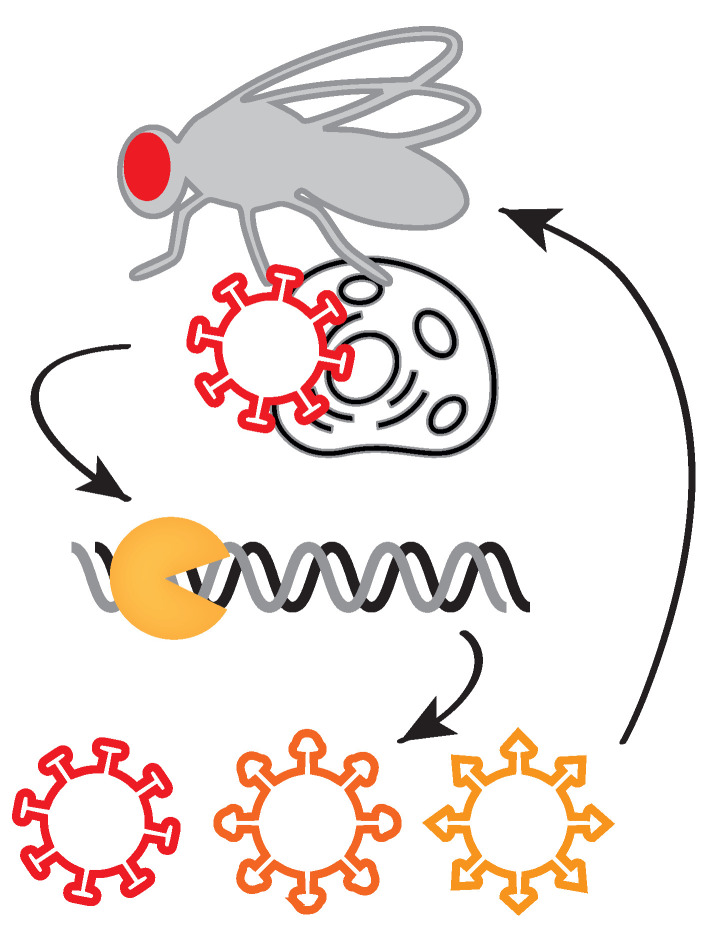
Viruses induce antiviral responses in their hosts, such as the RNA interference pathway in insects. Potent immune responses may drive the evolution of viral sequences and phenotypes.

**ESR-RUMC—Impact of host immune pathways on virus evolution** Evolution of viruses is strongly affected by antagonistic co-evolution of virus and host. Host immune pathways select for viruses that evade, avoid, or suppress the immune response, which in turn may drive counter-adaptations in host immune genes. For example, the antiviral RNA interference (RNAi) mechanism is actively suppressed by many insect viruses [19], likely inducing rapid evolution of RNAi genes of the host, as has been observed in the fruit fly *Drosophila melanogaster* and other invertebrates [20,21]. Whether such interactions exist for other immune pathways, such as Toll, IMD and Jak-Stat remains to be investigated. The overall aim of ESR-RUMC is to understand the impact of host antiviral responses on viral population dynamics (see Figure 8). For these studies, we will use natural virus-host combinations, including nora virus and *Drosophila* C virus, widespread pathogens of Drosophila that suppress RNAi at different stages [22,23].

**ESR-WZ—Elucidating the role of RNA modification during cytomegalovirus infection.** N6-methyladenosine (m6A) plays critical roles in RNA metabolism and function. In addition to the internal m6A, N6,2′-O-dimethyladenosine (m6Am) is present at the transcription start nucleotide of capped mRNAs. The methyltransferase that adds this modification, PCIF1, was recently identified but the functional importance of this modification still remained enigmatic.Human cytomegalovirus (HCMV) is an important human pathogen that replicates in the nucleus exploiting cellular machineries. We will investigate if HCMV utilises the m6Am modification by conducting m6A-IP and high throughput sequencing using primary fibroblasts and investigate its functional importance especially to viral titers in the absence of PCIF1. Our objectives are: (a) We will examine m6Am modification landscape during viral infection (on viral and host mRNA) by conducting m6A-IP on HCMV infected primary fibroblasts and analysing the enrichment of the first base compared to input control. (b) We will dissect the effect of this modification on viral propagation by generating PCIF1 KO cells. (c) We will analyse how m6Am modification affects viral RNA lifecycle using RNA-seq (total RNA), 4sU-seq (RNA production and RNA stability, JMU) and Ribo-seq (RNA translation) applied on infected PCIF1 KO and control cells.

### 3.4. Virus Regulation

Viruses circulate predominantly in their natural reservoir host but frequently also infect other species. However, to establish a new lineage the virus has to adapt to the new environment by acquiring advantageous new mutations. These mutations also influence the secondary structure of the viral DNA and RNA, in turn affecting the replication process. ESR-RKI, will study the fundamental correlation between virus evolution and RNA secondary structures. Being more specific, in case of segmented genomes (e.g., influenza A virus (IAV)) packaging is influenced by the secondary structure of the virus genomic RNA segments. In cells co-infected with different viruses, new variants with mixed genomes can emerge that have the potential to overcome the natural host species barrier and to cause a devastating pandemic. Mixing of viral segments, also designated genetic reassortment, is possible due to a very efficient incorporation of different genome segments into one virus particle. ESR-CNRS will systematically generate reassortant viruses between two human IAVs and select those that replicate most efficiently for further functional studies. Additionally, ESR-CNRS will use chemical probing to compare the genome structure in parental and those reassortant viruses. ESR-FSU will study genetic reassortment events of IAV and especially the role of RNA-RNA interaction that are supposed to coordinate the interaction of the different genomes which are finally incorporated into viral particles. The focus of ESR-FSU is to develop a computational tool that can predict the complex interplay of the RNA-RNA interaction required for genome packing between quite diverse IAV based on experimental data.

**Figure 9:**
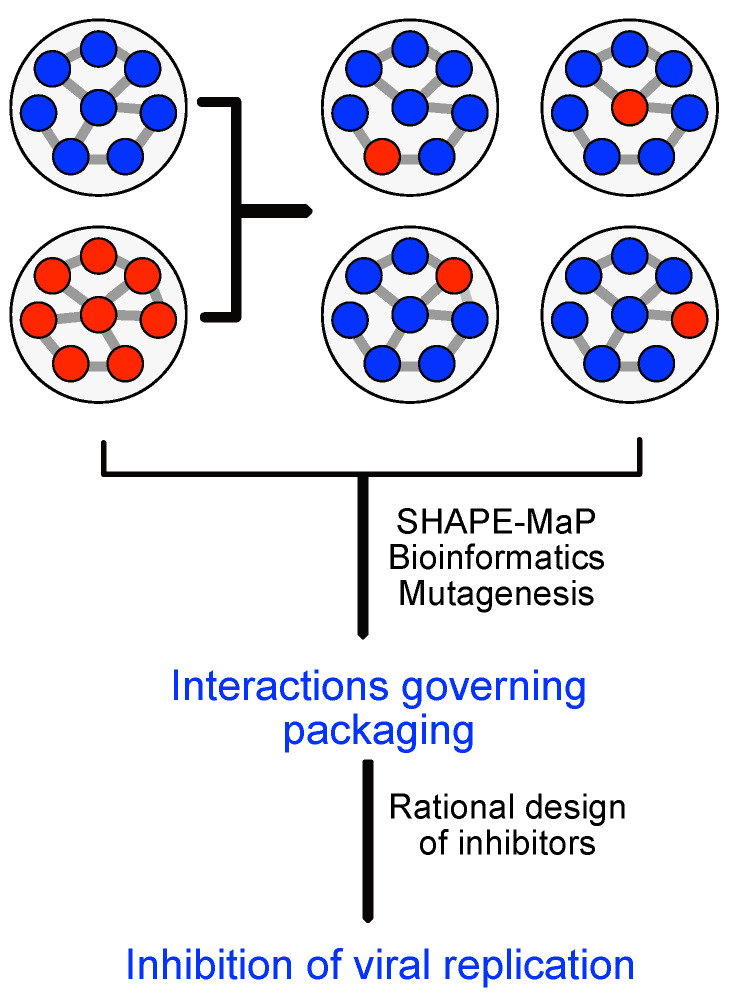
Preparation and analysis of re-assorted human influenca A virus to address the role of RNA-RNA interaction in genome packaging.

**ESR-FSU—Deciphering the RNA genome packaging code of influenza A viruses.** Currently, bioinformatical tools are not specifically designed for viruses. However, viruses bring unique features, which require specific bioinformatical tools to trace virus-host interaction. For example the number of sequences in a quasispecies is massively high due to their high mutation rate, but only a few interact again with the host cells. Some viruses, such as IAV, are segmented RNA viruses, which urgently require tools with specific features: RNA viruses should include standardised secondary structure predictions, leading to RNA-RNA interaction prediction necessary for the packaging of segmented RNA viruses, as preliminary depicted in [24,25]. Therefore the precise aims of this project are (a) Development of a bioinformatical tool to predict RNA-RNA interactions as packaging signal for segmented viruses, such as IAV. The tool will also consider the 3D arrangement of the RNA molecules being essential to understand the nature of packaged viruses; (b) Development of a virus specific full genome multiple sequence alignment algorithm to track the quasispecies. We will combine this tool with secondary structure information being available from literature and CNRS to build more accurate alignments; (c) Establishment of RNA-RNA interaction sets and more importantly non-interaction sets. The result will be used by CNRS to explore the reassortment space of IAVs (see Figure 9). This understanding is essential for a prediction of future Flu outbreaks; and (d) Application of the tool for at least 30 different viruses in other projects in VIROINF than CNRS (IAV).

**ESR-CNRS—Mechanism of coordinated incorporation of segmented virus genomes into viral particles.** Genetic reassortment between divergent IAVs in the host is a highly biased process in which only a small fraction of all possible genotypes are efficiently produced. This restriction is due to the limited compatibility between packaging signals of divergent IAVs and the limited conservation of RNA-RNA interactions required for genome packaging, but might also be influenced by the host as environment. Thus, analyses of genetic reassortment not only shed light on how viruses with pandemic potential are produced, but also provides crucial information about the coordinated incorporation of the segmented IAV genome. Therefore, we will (a) generate reassortants between two human IAVs that will contain seven segments from one parental virus and one segment from the second parental virus. (b) Compare the secondary structure of the genomic RNA segments in the two parental and up to eight selected reassortant viruses grown in human cells using chemical probing. This analysis will be performed with ESR FU1 and highlights differences in RNA-RNA interactions between parental and reassortant viruses (see Figure 9). These experimental data will be used by FSU to predict packaging signals. We will next (c) experimentally verify ESR-FSU packaging predictions. Finally, we will (d) generate antivirally active biochemical substances that specifically interfere with RNA-RNA interactions between genomes.

### 3.5. Virus Products

Currently, the area of enzymatic manipulation of nucleic acids is expanding in several different directions. VIROINF will be timely by using available viral metagenome data to explore the great potential to search for new enzymes useful in biotechnology. New viral enzymes have a high chance of showing new and different properties that can open up new or improved applications and expand the market. In particular thermophilic phages infecting bacteria and archaea have been recognised as a novel source for nucleic acid modifying enzymes. In most DNA/RNA applications high temperature gives a clear advantage and therefore a preference will be to search for enzymes from thermophilic (or other extremophilic) metagenomics samples. We aim for large-scale identification of phage sequences, functional annotation and in vitro validation of phages as treatment against harmful bacteria.

**ESR-UZH—Generation of experimental datasets for phage-bacterial interactions in complex communities and mutational spectra analysis following exposure to selective pressure to support artificial intelligence applications for R&D of bacteriophage products.** Bacteriophages are the most abundant organisms on Earth, making it difficult to select which ones have potential for therapeutic or other commercial applications. Current methods rely on non-iterative in vitro elaboration for product development, requiring >6 months per phage product. In the current project, we aim to generate input data from experiments to build deep learning algorithms that will guide and support phage product development by (a) revealing which phages compete best under which specific conditions and combinations, (b) identifying mutations important for phage activity and survival under environmental conditions (pH, temperature), and (c) demonstrating the population effects of phage infection in complex community structures. In this project, we will use a top-down approach to characterise the resulting community composition and mutational spectra from in vitro assays and/or clinical samples to measure: (i) competition between multiple phages; (ii) phage-interaction in complex communities to simulate microbiome and polymicrobial infection settings; and (iii) exposition of phage to multiple environmental conditions using genomic re-sequencing and/or cycling temperature capillary electrophoresis (CTCE) to identify regions of mutations associated with specific stimuli.

**Figure 10:**
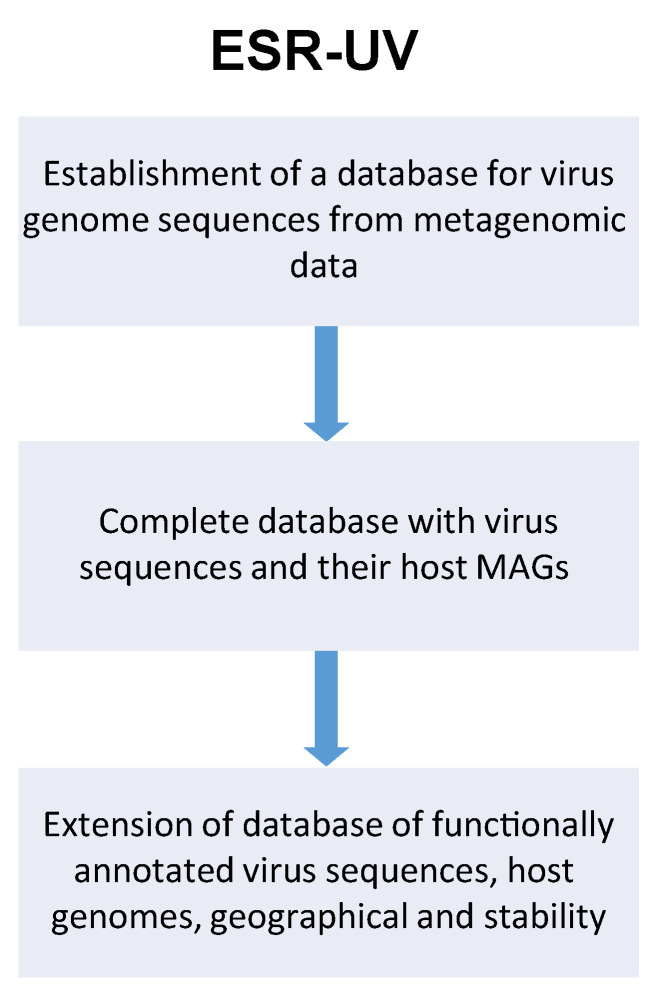
Schematic flow of ESR-UV project for establishment of virus metagenomic database.

**ESR-UV—Computational methods for the analysis of metagenomic datasets to extract viral sequences within the context of commercial data-mining/R&D support for bacteriophage products.** Bacteriophages are increasingly being developed for the treatment of bacterial infections, and their role as modulators of the microbiome is of a growing area of extension of phage use. The sheer number and diversity of phage particles makes it impossible to isolate and test each of them individually. This project aims to identify and harness phage sequences for commercial use by developing a method for large-scale identification of phage sequences, functional annotation, and in vitro validation of findings in a bottom-up approach. We have four specific aims: (i) Identification of phage sequences within >50 metagenomic datasets (including upstream generation of >5 metagenomic datasets by partner UZH and meta-analysis of >45 publicly available datasets). Reconstruction of viral genomes sequences from metagenomic data (assembly, binning, phage classification); Reconstruction of cellular genome sequences (MAGs) from the same/related metagenomic datasets, in order to extract potential phage-host pairs from co-occurrence patterns; Prediction of prophage sequences in MAG in order to facilitate host prediction of phage sequences (in collaboration with ESR-UMG). (ii) Extraction and testing of sequences to validate WP 1 findings ie., hypothesis testing by either isolating the phages themselves or having their genome synthesised (in collaboration with ESR-UZH). (iii) Sub-analysis of geographical and temporal variation in metagenomic datasets that could justify product composition or modification analysis of geographic and temporal variation of phage sequences according to the local epidemiology (see Figure 10). Relating these changes to geographic and temporal variation of related microbiome data (in collaboration with ESR-UZH). (iv) Select for sequences with developmental potential (phage sequences with greater pH stability; host range expansion, etc.); Computational modelling of phage stability and host range (in collaboration with ESR-UZH).

## 4. Modelling Virus Evolution in Hosts

We aim at deciphering evolutionary forces shaping viral adaptation at VIROINF. To this end, virus evolution experiments will be performed in the lab, generating vast amounts of genomic and transcriptomic data. Sophisticated bioinformatic tools will be developed that allow us to recover the evolutionary dynamics and relate phenotypic attributes to evolved genetic material, to changes in the transcriptome structure and in the viral quasispecies composition. VIROINF aims to develop tools for both established levels: In the area of microevolution, the short term evolution (at most a few years) is examined in the viral population, also known as ‘virus quasispecies’. In the area of macroevolution, the long term evolution (millions of years) is examined to determine the relation of virus families.

### 4.1. Microevolution: Virus Quasispecies

Viruses display high genetic diversity both within and among viral species, as well as within and among infected hosts. The composition of mixed samples can be assessed by metagenomics approaches [26], such as sequence read annotation by taxonomic classification using existing reference genomes and databases [27,28]. Such data will be generated within VIROINF. For the majority of novel viral sequences encountered in biodiversity studies no reference genome or homolog is known [29], and discovery of de novo viral species by state-of-the-art genome assemblers ignores low-frequency variants and technical errors. Low-frequency variants are of especially great interest for harbouring drug resistance mutations [30] or affecting virulence. In this context, with the true interdisciplinary approach of VIROINF the following questions can be answered: (i) How can we distinguish viral haplotypes in RNA-Seq data and characterise sequence-based evolution? (ii) What is the role of quasispecies in virus pathogenesis and evolution? (iii) How does intra-viral and viral-host selective pressure shape short-term evolution? (iv) Are RNA modifications also dictating some selective pressure? (v) Can we predict and design the fittest virus within a viral quasispecies? These five questions will be mainly addressed through the integration of virus evolution experiments that generate high-resolution 2nd and 3rd generation sequences and the development of novel bioinformatic tools to resolve quasispecies structures from the resulting data.

VIROINF will search for patterns which can emerge when host affiliation is projected onto viral taxonomy. Branch permutation techniques will be used to statistically determine at which level the viruses and hosts co-evolve. These patterns will reveal the evolution of virus-host associations for different classes of viruses. The associations will be validated by analysing both, known and predicted, host-associations independently. Moreover, virus-host associations will be used to validate the co-evolutionary signal.

**ESR-ETH—Development of new computational methods for characterisation and analysis of intra-host viral populations.** Viruses exist in their hosts as populations of genetically heterogeneous particles, often referred to as viral quasispecies. Intra-host genomic diversity can result in phenotypic heterogeneity, and it has been linked to viral pathogenesis and virulence. NGS can be used to assess the genomic heterogeneity of virus populations in a cost-efficient manner, but this approach is challenging due to sequencing errors and short read length. In this project, we will develop improved tools for the reconstruction of viral genomic diversity from both short-read and long-read NGS data, and we will leverage the power of the inferred population structure to inform models of virus micro-evolution, to detect selection, and to correlate population features to viral and host phenotypes.

**Figure 11:**
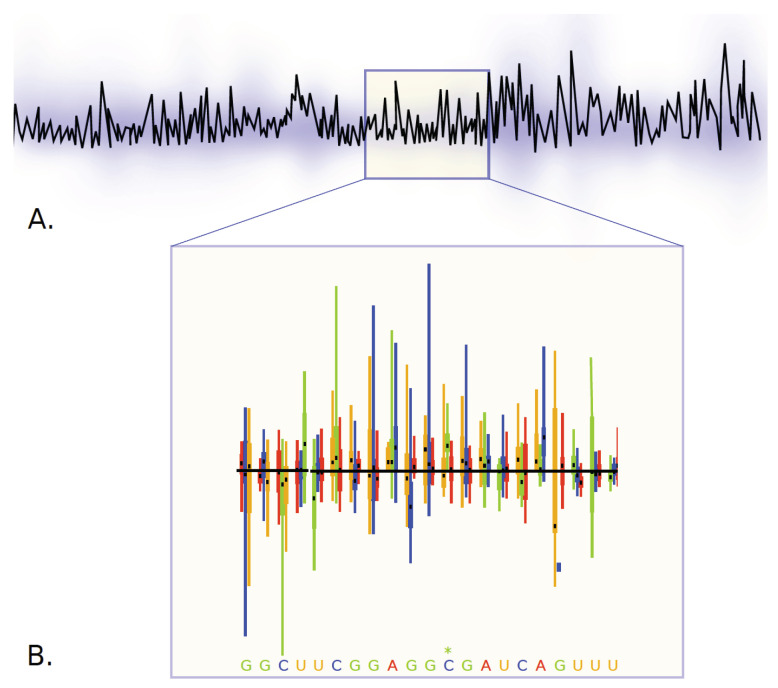
Viral genomes are usually very compact and harbor several coding and non-coding regions that are necessary to hijack host-cells. The team of ESR 8 uses Mutational Interference Mapping Experiments (MIME; [31,32]) to identify and study the function of non-coding regions in viral RNA genomes (ncRNA function). MIME generates millions of randomly mutated viral RNA genomes, which are subjected to selective pressure (for example: binding to initiating (80S) ribosomes = translation initiation). Following mutagenesis, pools of functionally selected vs. deselected RNA are physically separated and deep sequenced. The abundance of mutations at each position in functionally selected- and deselected RNAs is mathematically analyzed and allows to infer the phenotype of each mutation with regards to the analyzed function. This allows to identify functional domains in the RNA (peaks in panel (**A**)), as well as quantifying the phenotype of each mutation at each position of the RNA (boxplots in panel (**B**)).

**ESR-RKI—Genotype-Phenotype mapping and inference of epistatic interactions driving adaptation in viral hosts.** Viruses hijack the host cellular machinery for replication. This hijacking is driven by the interaction of viral proteins and non-coding RNAs with host-cellular components. Viral genomic sites instrumental to these interactions are thus conserved through evolution, yet bioinformatics analysis of evolutionary conservation cannot tell apart and quantify the functional relevance of genomic sites under selection. We have previously shown using the Mutational Interference Mapping Experiment (MIME) that in vitro [31] and in vivo [32] evolution experiments with subsequent NGS generate data sets that allow quantifying the phenotype of every nucleotide in a single experiment (see Figure 11). The goal of this project is to improve and tailor mathematical and computational methods [33] to identify and functionally characterise domains in the viral genome under evolutionary selection and to apply these tools to data from ESR-FU, ESR-RUMC, ESR-CNRS and our secondment.

On the technical side, (a) we will learn maximum entropy models (direct coupling analysis, DCA) from nucleotide abundances in functionally selected and de-selected virus quasispecies to identify single- and interacting sites. (b) As an alternative approach and a means to validate a), we investigate the application of deep learning approaches to identify higher-order epistasis in collaboration with secondment Bernhard Renard (HPI). (c) We then derive algebraic expressions from kinetic modelling of the investigated selection process to interpret the derived coupling terms mechanistically (phenotypically). This allows to characterise the complex (and possibly highly constraining) fitness landscape on which adaptation takes place. In the development phase our computational methods will be benchmarked by simulating the selection experiments and the corresponding NGS data sets, as well as with existing NGS datasets and phenotypic endpoints from previous work (e.g., HIV genome packaging). Subsequently, we will apply the tools to NGS data generated by in vitro evolution experiments of ESR-CNRS/secondment R. Smyth (HIRI) to investigate domains in the influenza genomic RNA, which are responsible for viral packaging/re-assortment, in collaboration with AllGenetics (AG). Moreover, we will apply the methods to in vivo evolution experiments conducted by ESR-FU (deformed wing virus), as well as ESR-RUMC (*Drosophila* virus) to predict phenotypic contributions to virus growth, mortality and host transcriptional responses.

### 4.2. Natural Selection of Viruses

Phylogenetic trees are the most widespread presentation for viral phylogenies in the literature. Several tree-building methods and software tools exist (e.g., MrBayes [34], RAxML [35]), but these methods produce incorrect results for viral phylogenies due to the complex evolutionary relationships that are relevant for viruses, such as horizontal gene transfer, intra- or interspecific recombination, positive selection, or the evolutionary relationships between viruses and their hosts. There is a clear need for specialised computational methods to support reconstruction of these special aspects of virus phylogeny. VIROINF finally combines the results of all ESR to contribute to the macroevolution of viruses. Existing phylogenetic methods assume that distinct sites within the genome evolve independently, which results in severe errors when evolution is driven by epistatic constraints. ESR-RKI, will infer the evolutionary linkages between distinct genomic sites in order to develop methods to accurately reconstruct viral phylogenies in the presence of disruptive processes such as virus recombination. The necessary genotypic data for this task will be derived from controlled evolution experiments carried out by ESR-FU, who will serially passage single and mixed viral genotypes (DWV-A and -B) in vivo (in the honeybee) to investigate the role of recombination in virus adaptation. The honeybee is a key model in environmental research and agriculture and the ESR-FU in this project will characterise how DWV evolves to exploit the honeybee host to increase virus replication, with a focus on characterising host transcriptome targets, including RNAi machinery and the Toll immune pathway (e.g., the key gene regulator dorsal). Virus phenotypic measures will include virus growth and virulence (host mortality) and will be matched against in-depth virus population sequencing in order to facilitate genotype-phenotype mapping with ESR-RKI. For large evolutionary distances, it is possible to infer phylogenies based on comparative analyses of macromolecular structures, which evolve much slower than sequences. This approach will be used by ESR-UZH and ESR-UV to compute phages serving as specific treatments against harmful bacteria.

**ESR-FU—Evolution of Deformed wing virus (DWV) in bees.**

**Figure 12:**
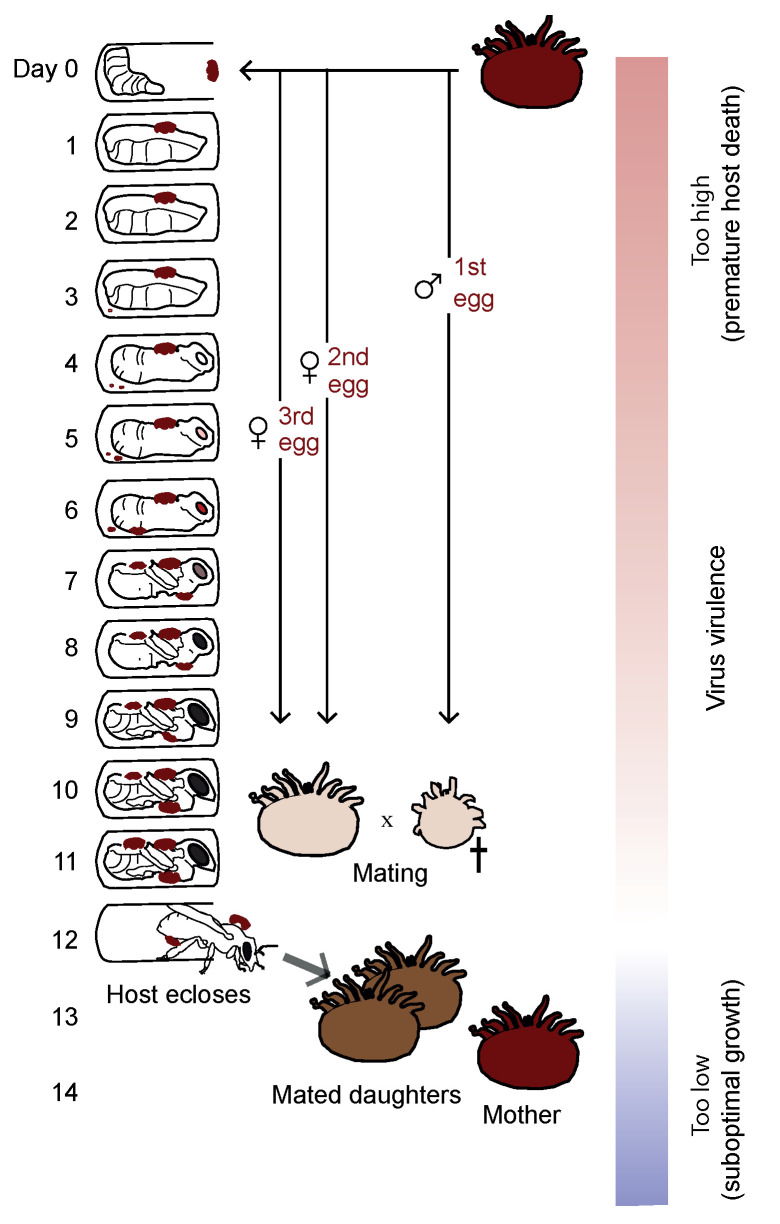
We will test the hypothesis that virus virulence evolves to maximize transmission (white region). Reproduced with permission from [36].

This project seeks to understand the evolution and impact of virus virulence. The honeybee virus Deformed wing virus (DWV) is an emerging pathogen that is responsible for widespread declines of honeybees in the Northern hemisphere. The impact of the virus has been exacerbated by the arrival of an ectoparasitic mite: *Varroa destructor*, which can vector the virus. In this project, we will carry out experiments with the aim of understanding the genomic basis of virus virulence (see Figure 12). Together, our study aims to help to understand the drivers of virus spread and damage in honey bee hosts that are of major economic and ecological importance.

## 5. Training

**Figure 13:**
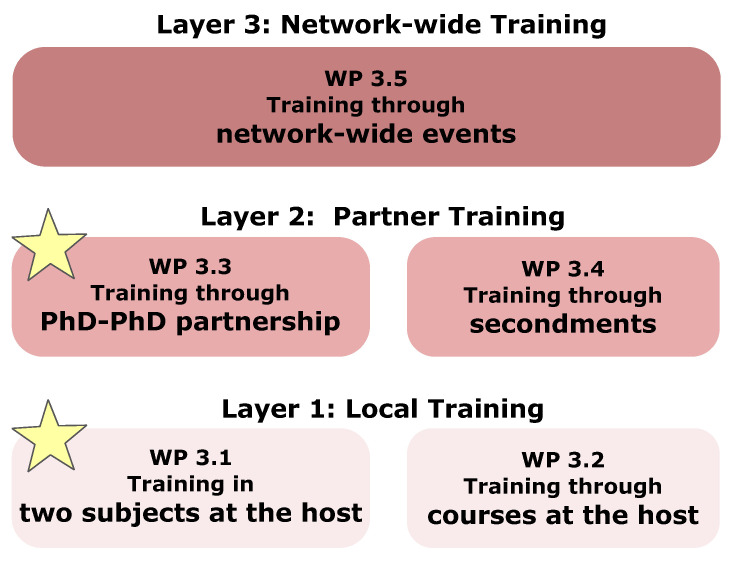
Three-layer-concept for intensive training in VIROINF.

The overall structure of the VIROINF training programme contains three main layers: **Layer 1:** project specific training (bioinformatics and virology) and scientific and transferable skill courses at the host institution; **Layer 2:** training through project-specific ESR-ESR partnerships (one being a virologist, the other being a bioinformatician), and through secondments; **Layer 3:** training through network-wide events. Yellow stars indicate additional training for the ESRs (see Figure 13).

## 6. Conclusions

The research programme presented addresses bioinformatical and virological challenges in the analysis of massive “big data” generated by new genome sequencing technologies. New tools offer, for the first time, the possibility to answer fundamental questions in virology, especially in virus-host biology. We emphasise that many of the questions raised in virology require specific bioinformatics support and the combined expertise of many groups of both bioinformaticians and virologists. The strategy of the network is therefore to develop parallel lines of bioinformatics research to address a number of specific key questions in viral research.

We hope to be a nucleus that not only conducts fundamental research, but also reaches out into both research communities—virologists and bioinformaticians—to foster further collaborations. We are open to discussions about integrating your appreciated research in any way possible, from joint talks or conferences, to pair-wise collaborations, to association in our consortium. Please do not hesitate to contact us (https://viroinf.eu (accessed on 26 April 2021)).
